# Annotations

**Published:** 1888-10-13

**Authors:** 


### Annotations.

**A Defender of Women.**

Mrs. Fenwick Miller has taken advantage of the recent movement of public opinion to advocate the claims of women to more consideration at the hands of the law. She avers that offences against property are much more severely dealt with than offences against the person, especially if the person happens to be a woman. A long list of cases is brought forward in support of Mrs. Fenwick Miller's position, and they are sufficient to make it evident that there is a good deal of point in her contention. It is not impossible that the special pleading of Mrs. Miller overlooks one part of the case. The abuse of women by men is no doubt very gross and very brutal; but the fault, though largely, is not entirely on the side of the men. It consists with our experience that women of the lower class who give way to drink and bad habits, are often insanely provoking, and cannot be kept in any kind of order by gentle measures. One woman known to the writer has so often given way to drink, and when under the influence of it has been so mad, and caused such tremendous upset in the house and the neighbourhood that her husband has been simply a miracle of patience, in that he has kept his hands off her. Many a time an onlooker would have declared that a sound and effectual thrashing was the only possible remedy. We are not advising men to thrash women by any means. Be a woman never so provoking, there can be no good reason why physical force should be used. But we would ask Mrs. Miller to bear in mind that many a time a moderate sentence for a heavy offence may be the result of the magistrate's knowledge of extenuating circumstances—knowledge which is not furnished by the newspaper report of the case. The subject is one of deep importance, not so much as demanding a change of the law in the direction of severity, but as showing how brutalised the lower classes had to become when all relegated to one quarter of the metropolis or other large towns and cut off from association with any other class. The social system, which is so ordered as to make sharp lines of demarcation between the rich and the poor, the civilised and the brutal, is at fault. It is difficult to suggest any remedy for the fundamental failure of our own civilisation, but it is certain that brutality among men will never be cured by severity of laws, but only by the bringing about of a better state of morals and behaviour both among men and women. The problem is to be solved by improved social and economic conditions, not by increased severity of the penal code.

**Fallen Women.**

The recent tragedies in Whitechapel have for a brief period lifted the veil which ordinarily hides from the world the life and doings of fallen women. Shocking and incredible as it may seem, the bulk of such women come to look upon their avocation as work done for bread, exactly the same as other people do. The picture disclosed to our view is often that of a middle-aged woman, once respectable, the child of respectable parents, the sister of self-respecting sisters and brothers, in some instances the mother of virtuous and industrious daughters. This woman, sunk below the level of animals in the foul uncleanness of her way of life, in her mad craving for drink, in her self-abandonment to every impulse which circumstances permit her to indulge, is for all practical purposes a moral and mental ruin, and is soon to become as complete a ruin physically as she is already morally. But the saddest and most heartbreaking of all the facts of her history is this, that she still in many instances preserves her moral susceptibilities. As one witness said, in speaking of her fallen sister: "We seldom saw each other, because our way of life was different; but when we did my sister always cried, and always wished she could be as I was but she could not." The pity of it is overwhelming: to have eyes to see that one is a wreck and a ruin, but to have no power even to begin a better way of life. An experienced clergyman says of these women: "After a time they are practically hopeless. Their wants are very few. Food hardly seems to be a necessary with them. A bed to sleep in and plenty of beer seem to be all they really require. Even if they can be induced to enter a home, they are unaccustomed to the slightest restraint, and will submit to none. They become amazingly reckless." He gives an instance of two strong middle-aged women of this class. They were quarrelling. "One was down in the street and the other at an open window at the top of a house. They railed and stormed at each other for a time, and the one up at the window worked herself up to such a frenzy of passion that in her eagerness to get at her tormentor she actually flung herself down headlong at her, just as a wild beast might have sprung. There are many such women about who are practically past hope. They are a great terror to the police, for they are as strong as men, and constables can't tackle them as they do men. Being women, they can't hit them, but the women themselves fight desperately." There is no romance here; these are hard facts, of every-day occurrence. Even callous and cold-blooded men must feel that they are terrible facts, whilst those who believe in the higher characteristics and possibilities of humanity are paralysed with horror and despair. In the face of these and innumerable other evidences of widespread degradation and ruin among our fellow-men, it becomes all who are on the side of religion and virtue to close up their ranks, to put away all vain disputes and useless controversies, and to devote themselves to the one purpose of raising the social mass by means of principles and examples, until it shall become worthy of the name of man and of the light and knowledge which distinguish him from the brute.

**Malthus and the Vicar.**

The Vicar of Middlewich, the Rev. Francis Minton, M.A., is a heretic of a remarkable kind. In one and the same sentence he expresses the strongest possible dissent from two such learned and authoritative bodies as the British Association and the Church Congress. The representatives of science and those of religion he considers to be equally in the wrong. Mr. Minton, indeed, appears to understand, as a few other people understand, that there is no particular importance to be attached to the corporate opinions of the two assemblies in question, and that for the very good reason that both British Associations and Church Congresses are just as ready to follow any self-confident bell-wether as any other large gathering of human beings. When, therefore, the speakers at the recent meetings trotted out once more the exploded theories of Malthus, and were echoed as to their sentiments by a chorus of "Amens" by both assemblies, Mr. Minton did not join in the "Amens." He consoled himself with the reflection that, after all, the opinions expressed had only been thought out by one or two, whilst the great multitude followed because they believed they were confirming an accepted dictum of the times. Mr. Minton has carefully considered the "over-population" question for many years. His practical conclusions are—that it will be several centuries before the world can be as thickly populated as Belgium; that a large community is capable of producing more food and wealth—man for man—than a small one; that it is the tendency of poverty, more than of comfort, to increase population—which is the exact reverse of Malthus' opinion: that instead of nature utilising vice and misery to keep down populations, she stimulates the increase of populations to keep down vice and misery; that modern statistics silence Malthus with crushing force. Mr. Minton points out that Professor Thorold Rogers, of Oxford, "the greatest living authority on democratic economics," has rejected the doctrines of Malthus; and that the most eminent of modern statisticians, including Mulhall, Giffen, Leone Levi, and others, incontrovertibly prove the most important point of the whole contention, viz., that man for man, a large community is capable of producing more food and wealth than a small one. What, then, is the practical conclusion? Mr Minton probably knows it very well: The land must be put to its utmost producing capacity. It must be made fully available for the general use of the people. There is in Mr. Minton's own county an estate owned by a Conservative landlord. It is divided into farms of all sizes, except very large ones. It has numerous small plots for labourers. Throughout the past ten years of depression, both landlord, tenants, and labourers on that particular estate have been prosperous. What does the example signify? This: that the remedy for agricultural, and with it most other kinds of depression, is in our own hands. Malthusianism is a mere quack specific; the free use of the land would be a rational and scientific cure.

